# The impact of postoperative ischemic changes on survival outcomes in IDH-wildtype glioblastoma

**DOI:** 10.1093/noajnl/vdaf235

**Published:** 2025-11-20

**Authors:** Neslihan Nisa Gecici, Ahmed Habib, Walaa Hamza, Allyson Andrews, Rivka R Colen, Sameer Agnihotri, Pascal O Zinn

**Affiliations:** Department of Neurological Surgery, University of Pittsburgh Medical Center, Pittsburgh; Department of Neurological Surgery, University of Pittsburgh Medical Center, Pittsburgh; Department of Radiology, University of Pittsburgh Medical Center, Pittsburgh; Department of Neurological Surgery, University of Pittsburgh Medical Center, Pittsburgh; Department of Radiology, University of Pittsburgh Medical Center, Pittsburgh; Department of Neurological Surgery, University of Pittsburgh Medical Center, Pittsburgh; Department of Neurological Surgery, University of Pittsburgh Medical Center, Pittsburgh

**Keywords:** deficit, diffusion restriction, glioma, infarct, ischemia, survival

## Abstract

**Abstract:**

BackgroundGlioblastoma (GBM) is an aggressive brain tumor. The authors of this study wanted to find out whether areas of reduced blood flow, called ischemia, that appear after surgery affect how long people live with this disease. To do this, they reviewed scans from 451 patients taken within three days after tumor removal and measured the size of any new areas with poor blood supply. Their results showed that larger areas of ischemia were linked to shorter survival. The prognostic relevance of postoperative ischemia in GBM remains unclear. This study investigated the association between infarct volume and survival in patients with Isocitrate Dehydrogenase (IDH)-wildtype GBM.

**Methods:**

We retrospectively reviewed 451 patients with IDH-wildtype GBM who underwent resection between 2013 and 2024 and had diffusion-weighted imaging within 72 h postoperatively. Ischemic changes were defined as new areas of diffusion restriction and stratified into none/rim-only, small (<5 cm³), and large (≥5 cm³) infarcts. Progression-free survival (PFS) and overall survival (OS) were analyzed using Kaplan–Meier and Cox regression models adjusted for age, preoperative KPS, tumor size, extent of resection, MGMT status, adjuvant therapy, and postoperative deficits.

**Results:**

Large infarcts were associated with shorter median PFS (7.0 months [95% CI: 5-9]) and OS (14.0 months [95% CI: 9-18]) compared to small infarcts and none/rim-only (PFS: *P* = .07; OS: *P* = .001). In multivariable models, infarct volume was independently associated with reduced OS (per cubic centimeter increase, HR = 1.02, 95% CI: 1.01-1.032; *P* = .003), while its association with PFS did not reach statistical significance (HR = 1.01, 95% CI: 1.0-1.02; *P* = .13). When modeled categorically, large infarcts remained predictive of shorter PFS (HR = 1.4, 95% CI: 1.01-1.9; *P* = .04) and OS (HR = 1.7, 95% CI: 1.2-2.4; *P* = .001).

**Conclusions:**

Infarct volume is independently associated with survival in IDH-wildtype GBM. These findings highlight the clinical relevance of postoperative ischemia and may point toward ischemia-related mechanisms as targets for future therapeutic investigation.

Key Points

- Infarct volume is independently associated with survival.
- This relationship persists after accounting for postoperative neurological deficits.
- Findings suggest a potential role of ischemia in glioblastoma progression.

Importance of the study

Perioperative ischemia is frequently observed following glioblastoma resection, yet its prognostic implications remain poorly defined. This study examines the association between postoperative ischemic changes and survival in a large, molecularly homogeneous cohort of patients with IDH-wildtype glioblastoma. Our findings demonstrate that infarct volume is independently associated with poorer survival, regardless of postoperative neurological deficits. These results suggest that ischemic injury may contribute to glioblastoma progression through mechanisms beyond functional decline, potentially involving hypoxia-driven biological processes. Recognizing infarct burden as a modifiable risk factor may inform surgical decision-making and highlight avenues for future translational research.

Glioblastoma (GBM), the most aggressive malignant glial tumor in adults, has a poor prognosis despite advances in understanding its biology and improvements in treatment approaches, with a median survival of 9 months untreated and 17 months with intervention.^1–3^ Maximal resection with preservation of neurological functions remains the goal of surgical treatment as postoperative transient and permanent deficits have been shown to be associated with decreased survival.^4–7^ Such deficits may result from direct tissue injury during resection or secondary complications such as hemorrhage, venous congestive infarcts, and arterial ischemic events.^7^^,^^8^

Perioperative ischemic changes occur in approximately 25% to 70% of glioma surgeries and are most commonly identified on postoperative imaging, as intraoperative neuromonitoring and MRI have demonstrated high false-negative rates.^4^ These ischemic changes typically present as new diffusion restrictions, appearing as hyperintensities on diffusion-weighted imaging (DWI) with corresponding hypointensities on apparent diffusion coefficient (ADC) maps.^4^^,^^7–14^ Several studies have demonstrated a significant correlation between these changes and neurological deficits, and infarcts leading to functional decline have also been associated with reduced survival in glioma patients.^4^^,^^7^^,^^8^^,^^12^^,^^14^ Additionally, some studies have reported that larger infarct volume is independently associated with worse overall survival (OS) and more aggressive recurrence patterns, though more recent studies have not confirmed these associations.^7^^,^^11^^,^^15–17^

This study aimed to evaluate the impact of postoperative ischemic changes on survival outcomes in IDH-wildtype GBM. We additionally examined their association with neurological deficits at discharge and accounted for their influence in multivariable-adjusted analyses.

## Methods

### Study Design and Patient Selection

This study is approved by the Institutional Review Board and requirement for formal patient consent was waived. The study included patients with IDH-wildtype GBM, treated at the University of Pittsburgh Medical Center from January 2013 to June 2024, with follow-up until February 2025. Patients without postoperative DWI imaging within 72 h, those without follow-up data, and those who underwent biopsies only were excluded.

### Data Collection

Patient and tumor characteristics, as well as operative and outcome data, were collected retrospectively from electronic medical records. IDH status was evaluated by immunohistochemical staining and/or next-generation sequencing (NGS). New or worsened neurological deficits were defined as any postoperative decline in motor, sensory, speech, or coordination function observed at discharge, as assessed by the attending physician during the follow-up visit.

Postoperative ischemia was identified on DWI as a new diffusion restriction in the brain parenchyma compared to preoperative imaging, defined by hyperintensity on the B1000 and B2000 series and hypointensity on the ADC series. The infarct volume was assessed using region of interest (ROI)-based volumetry. For this measurement, the entire ischemic region was identified and traced across all postoperative DWI axial imaging slices. A 3D ROI-based volume was then calculated by summing the ischemic areas on each slice and multiplying by the slice profile. Postoperative changes, including edema and methemoglobin, were excluded from these volumes by assessing T2-weighted FLAIR and gradient-echo sequences. As small areas of diffusion restriction are commonly observed around the resection cavity postoperatively, a 3-mm radial cut-off was applied to distinguish these changes, which were defined as rim-restriction based on previous studies.^12^^,^^18^ The extent of resection (EOR) was classified as gross-total resection (GTR) and subtotal resection (STR) based on preoperative and postoperative MRIs, where any noted residual enhancement abnormality consistent with residual tumor was considered to be STR. A radiologist independently reviewed preoperative and postoperative images and was blinded to patient outcomes.

OS was measured from the date of surgery until death or the last follow-up, while progression-free survival (PFS) was measured from surgery until disease progression or recurrence. Disease progression or recurrence was determined only after a multidisciplinary review by neurosurgeons, neuro-oncologists, neuroradiologists, and radiation oncologists who assessed both imaging findings and the clinical course. Additionally, where applicable, advanced imaging modalities such as perfusion MRI and MR spectroscopy were used to support this evaluation.

### Statistical Analysis

Categorical variables were reported as numbers and percentages, whereas continuous variables were presented as mean and standard deviation for normally distributed data and as median and interquartile range for nonnormally distributed data. Comparative analyses were conducted using the *t*-test for continuous variables and χ^2^ or Fisher’s exact test for categorical variables, as appropriate.

Kaplan–Meier curves were constructed to estimate median OS and PFS, with comparisons between groups made using the log-rank test. A multivariable Cox proportional hazards model was utilized to investigate the impact of postoperative ischemia on OS and PFS, after adjusting for age, preoperative Karnofsky Performance Scale (KPS) score, tumor size, EOR, MGMT methylation status, chemoradiation, and new or worsened neurological deficit at discharge. The proportional hazards assumption test was tested by plotting the scaled Schoenfeld residuals against time and computing p-value for variables included in the models. Multicollinearity among variables was tested by calculating variance inflation factors.

All statistical analyses were performed using RStudio (2023.12.1 + 412, R Foundation, Vienna, Austria). Graphs were created using GraphPad Prism v10.0 (GraphPad Software, Boston, Massachusetts, USA). Hazard ratios (HR) with 95% confidence intervals (CI) and *P*-values were reported for all regression analyses. A *P*-value of < .05 was considered statistically significant.

## Results

**Figure 1.**
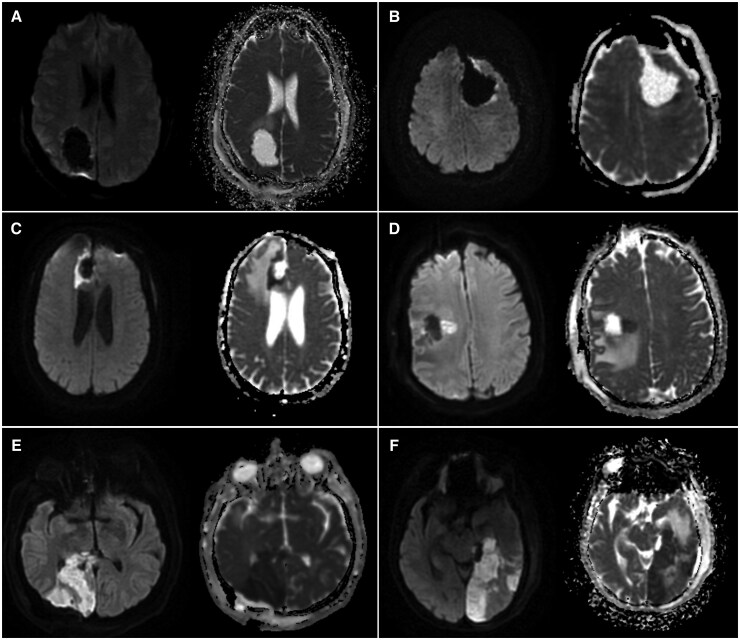
Postoperative diffusion-weighted imaging (DWI) and apparent diffusion coefficient (ADC) scans illustrating different patterns of ischemic changes: (A) no significant diffusion restriction, (B) rim restriction only, (C) small ischemic changes without new or worsened deficits, (D) small ischemic changes with a new postoperative motor deficit, (E) larger, confluent ischemic changes without new or worsened deficits, and (F) larger, confluent ischemic changes with a new postoperative visual deficit.

A total of 451 patients were included in the study, and a summary of patient characteristics is shown in Table 1. The mean age was 62.9 ± 11.3 years, with 42.8% (*n* = 193) being female. Most of the cases were newly diagnosed (*n* = 431, 95.6%), while twenty cases were recurrent GBM (4.4%). Postoperative ischemic changes were present in 190 patients (42.1%) (Figure 1). Of these, 96 (21.3%) were limited to the rim of the resection cavity. The median infarct volume was 5.4 cm³ [IQR: 2.7-9.5 cm³]. While the effect of infarct volume on survival outcomes will also be evaluated as a continuous variable in this study, to better visualize the differences between patients with and without ischemic changes, patients were grouped as follows: no ischemic change or rim restriction only (n = 357, 79.2%), small ischemic changes (< 5 cm³, *n* = 45, 9.9%), and large, confluent ischemic changes (≥ 5 cm³, *n* = 49, 10.9%).

**Table 1.** Baseline characteristics

		Ischemic changes			
Variable	Total	None or rim restriction only	Small DWI changes (<5 cm^3^)	Larger, confluent DWI changes (≥ 5 cm³)	*P* value
N of patients	451 (100)	357 (79.2)	45 (9.9)	49 (10.9)	
Age	62.9 ± 11.25	62.6 ± 11.36	63.9 ± 10.1	65.3 ± 10.3	.236
Gender					.515
Male	258 (57.2)	204 (57.1)	23 (51.1)	31 (63.3)	
Female	193 (42.8)	153 (42.9)	22 (48.9)	18 (36.7)	
Preoperative KPS					.248
100-80	388 (86)	303 (84.9)	39 (86.7)	46 (93.9)	
≤70	63 (14)	54 (15.1)	6 (13.3)	3 (6.1)	
Preop deficits	244 (54.1)	197 (55.2)	22 (48.9)	25 (51.0)	.653
Motor <3/5	12 (2.7)	9 (2.5)	2 (4.4)	1 (2.0)	.573
Motor ≥3/5	90 (20)	77 (21.6)	7 (15.6)	6 (12.2)	.24
nsory	26 (5.8)	25 (7.0)	1 (2.2)	0 (0.0)	.07
Speech	99 (22)	73 (20.4)	13 (28.9)	13 (26.5)	.257
Facial droop	36 (8)	26 (7.3)	6 (13.3)	4 (8.2)	.336
Visual loss	9 (16.1)	60 (16.8)	6 (13.3)	8 (16.3)	.872
Smoking history					.461
Never smoker	276 (61.3)	217 (61.0)	29 (64.4)	30 (61.2)	
Active smoker	40 (8.9)	36 (10.1)	1 (2.2)	3 (6.1)	
Past smoker	134 (29.8)	103 (28.9)	15 (33.3)	16 (32.7)	
Tumor characteristics					
Location					
Frontal	167 (63)	139 (38.9)	15 (33.3)	13 (26.5)	.213
Parietal	150 (33.3)	122 (34.2)	12 (26.7)	16 (32.7)	.632
Temporal	214 (47.5)	154 (43.1)	28 (62.2)	32 (65.3)	**.001**
Occipital	68 (15.1)	50 (14.0)	9 (20.0)	9 (18.4)	.399
Periventricular	281 (62.3)	220 (61.6)	31 (68.9)	30 (61.2)	.679
Insular	84 (18.6)	54 (15.1)	13 (28.9)	17 (34.7)	**.001**
Basal ganglia	34 (7.5)	22 (6.2)	4 (8.9)	8 (16.3)	**.04**
Tumor diameter, cm	4.36 ± 1.56	4.3 ± 1.5	4.3 ± 1.6	5.01 ± 1.9	**.008**
IDH wildtype	451 (100)	357 (100)	45 (100)	49 (100)	
MGMT methylation	221 (49)	169 (47.3)	28 (62.2)	24 (49.0)	.175

Abbreviations: IDH, isocitrate dehydrogenase; KPS, Karnofsky Performance Scale; MGMT, O6-Methylguanine-DNA Methyltransferase.

Bold text indicates statistical significance.

Age, gender, preoperative KPS, and preoperative deficits were comparable across groups (*P* > .05). Smoking history did not differ significantly between groups (*P* = .461). Tumors involving temporal lobe (*P* = .001), insula (*P* = .001) and basal ganglia (*P* = .04) were significantly more common in patients with small or large infarcts compared to those without ischemic changes. Larger tumor diameter was associated with more extensive ischemic changes (*P* = .008, Table 1). Although the duration of surgery was longer in patients with large ischemic changes compared to those with small changes and without ischemia (234.9 ± 84.9 vs 207.2 ± 83.07 vs 212.44 ± 105.3 min, *P* = .639), the difference was not statistically significant. GTR was more frequently performed in patients with large ischemic changes (63.3%) compared to those with small ischemic changes (37.8%) and no or rim restriction only (46.2%) (*P* = .034).

### Postoperative Outcomes

New or worsened neurological deficits at discharge were more common in patients with large (46.9%) and small ischemic changes (26.7%) compared to those with no ischemia or rim restriction only (15.7%) (*P* < .001), with a similar difference observed for new or worsened motor deficits (30.6% in large [≥ 5 cm³] infarcts, 15.6% in small infarcts [<5 cm^3^], versus 9% in none/rim restriction only, *P* < .001) (Table 2). There was a moderate positive correlation between infarct volume and postoperative new or worsened neurological deficits (*r* = 0.294, *P* < .001). The presence of ischemic changes was also associated with a longer hospital stay, with patients in the large infarct group having the longest duration (5.6 ± 3.1 days), followed by the small infarct group (3.7 ± 2.3 days), compared to those with no ischemia or rim restriction only (4.2 ± 3.2 days) (*P* = .008). Similarly, the proportion of patients discharged to rehabilitation was highest in the large infarct group (61.2%), 35.6% in the small infarct group, and 26.3% in those with no ischemia or rim restriction only (*P* < .001). In contrast, there were no significant differences in the percentage of patients receiving concurrent chemoradiation (*P* = .698) or in the time to its initiation (*P* = .923) among groups. The incidence of multifocal and distant recurrences, as well as recurrences contacting the ventricles during follow-up, was comparable among groups (Table 2).

**Table 2.** Surgical and postoperative characteristics

		Ischemic changes			
Variable	Total	None or rim restriction only	Small DWI changes (<5 cm^3^)	Larger, confluent DWI changes (≥ 5 cm³)	*P* value
Duration of surgery, minutes	215.46 ± 99.23	212.44 ± 105.3	207.2 ± 83.07	234.9 ± 84.9	.639
Extent of resection					**.034**
GTR	213 (47.2)	165 (46.2)	17 (37.8)	31 (63.3)	
STR	238 (52.8)	192 (53.8)	28 (62.2)	18 (36.7)	
Postoperative characteristics					
DWI changes in eloquent areas	31 (6.9)	–	10 (22.2)	21 (42.9)	**<.001**
New or worsened deficits at discharge	91 (20.2)	56 (15.7)	12 (26.7)	23 (46.9)	**<.001**
New or worsened motor deficits at discharge	54 (12)	32 (9.0)	7 (15.6)	15 (30.6)	**.001**
Length of stay	4.3 ± 3.1	4.2 ± 3.2	3.7 ± 2.3	5.6 ± 3.1	**.008**
Discharge to rehab	140 (31)	94 (26.3)	16 (35.6)	30 (61.2)	**<.001**
Concurrent chemoradiation	436 (96.7)	346 (96.9)	43 (95.6)	47 (95.9)	.698
Time to chemoradiation, weeks	4.83 ± 1.6	4.85 ± 1.55	4.75 ± 2.13	4.85 ± 1.41	.923
Recurrence type					
Multifocal	149 (33)	114 (31.9)	19 (42.2)	16 (32.7)	.698
Contact to ventricle	253 (56.1)	203 (56.9)	27 (60.0)	23 (46.9)	.363
Distant	175 (38.8)	135 (37.8)	23 (51.1)	17 (34.7)	.187

Abbreviations: GTR, *gross-total resection*; STR, *subtotal resection*.Bold text indicates statistical significance.

### Univariable Associations between Postoperative Ischemic Changes and Survival Outcomes

**Figure 2.**
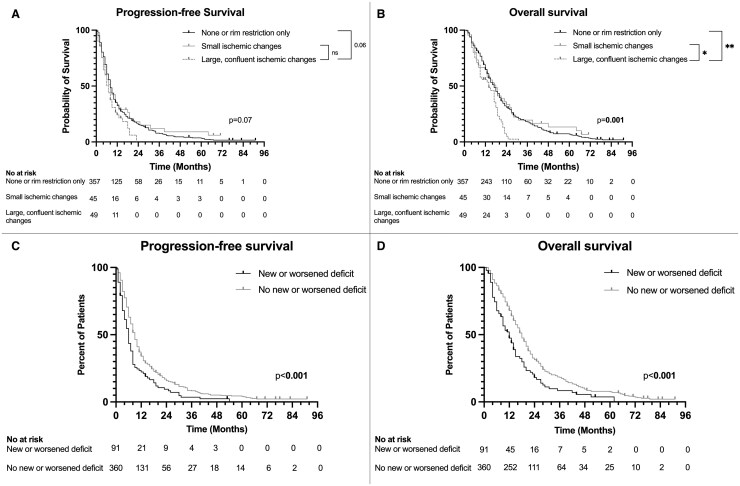
(A) Kaplan–Meier curve for progression-free survival (PFS) stratified by the severity of ischemic changes. Patients with large, confluent ischemic changes had PFS comparable to those with small ischemic changes (*P* = .31) and to those with none or rim restriction only (*P* = .06). (B) Kaplan–Meier curve for overall survival (OS) stratified by the severity of ischemic changes. Patients with large, confluent ischemic changes exhibited significantly worse OS compared to those with small ischemic changes (*P* = .02) and those with none or rim restriction (*P* = .001). (C) Kaplan–Meier curve for PFS based on the presence of new or worsened neurological deficits. Patients with new or worsened deficits had significantly shorter PFS compared to those without new or worsened deficits (*P* < .001). (D) Kaplan–Meier curve for OS based on the presence of new or worsened neurological deficits. Patients with new or worsened deficits had significantly worse OS compared to those without new or worsened deficits (*P* < .001).

The median PFS for the cohort was 8 months [95% CI: 7.2-8.8]. Kaplan–Meier analysis and pairwise comparisons showed that patients with large, confluent ischemic changes had comparable PFS (7 months [5-9]) compared to those with smaller ischemic changes (8 months [5-13] and no ischemia or rim restriction only (8 months [8-9]) (p [log-rank] = 0.07) (Figure 2A).

The median OS was 17 months [15.5-18.5]. Patients with large, confluent ischemic changes had significantly shorter OS (14 months [9-18]) compared to those with smaller ischemic changes (18 months [13-26]) and no ischemia or rim restriction only (17 months [15-19]) (*P* = .001) (Figure 2B).

### Univariable Associations between Postoperative Neurological Deficits and Survival Outcomes

Given the higher incidence of new or worsened neurological deficits among patients with postoperative ischemic changes compared to those without, the potential impact of these deficits on survival outcomes was further examined. Kaplan–Meier analysis revealed that patients with new or worsened deficits at discharge had significantly shorter PFS (6 months [4.9-7.2]) compared to those without new or worsened deficits (9 months [8.1-9.9]) (*P* < .001) (Figure 2C). A similar pattern was observed for OS, where patients with new or worsened deficits had significantly shorter survival (12 months [8.7-15.3]) compared to those without (18 months [16.5-19.5]) (*P* <.001) (Figure 2D).

### Adjusted Analyses Evaluating the Association between Infarct Volume, New or Worsened Deficits, and PFS and OS

Multivariable Cox regression analyses were subsequently performed to determine whether the univariable associations between infarct volume, new or worsened neurological deficits, and both progression-free and OS remained independent of one another and of other clinical confounders. Two separate models were used for both PFS and OS: one included infarct volume as a continuous variable, while the other categorized it into groups as previously defined. All models were adjusted for age, preoperative KPS, tumor size, EOR, MGMT methylation, and history of adjuvant chemoradiation.

When treated as a continuous variable, infarct volume demonstrated a small but gradually increasing association with the risk of progression (per cubic centimeter increase, HR = 1.01 [95% CI: 1.0-1.02], *P* = .1) and a statistically significant independent effect on the risk of death (per cubic centimeter increase, HR = 1.02 [95% CI: 1.01-1.032], *P* = .*P* = .003). New or worsened deficits at discharge were also independently associated with reduced PFS (1.4 [1.1-1.8], *P* = .009) and OS (1.3 [1.01-1.7], *P* = .04) in these models. In the other models where infarct volume treated as a categorical variable, only larger ischemic changes were found to be independently associated with shorter PFS (1.4 [1.01-1.9], *P* = .04), and OS (1.7 [1.2-2.4], *P* = .001) (Table 3). These findings suggest that while even small increases in infarct volume might contribute to shorter PFS and OS, the adverse effect becomes more evident at larger infarct volumes. The presence of new or worsened deficits was also independently associated with reduced PFS (1.4 [1.1-1.8], *P* = .01) and OS (1.3 [1.03-1.7], *P* = .03) in these models.

**Table 3.** Multivariable cox regression analysis for survival outcomes

	Progression-free survival			Overall survival		
Variable	Hazard ratio	95% CI	*P* value	Hazard ratio	95% CI	*P* value
Age						
≤65	Ref	Ref	Ref	Ref	Ref	Ref
>65	1.26	1.04-1.53	**.02**	1.48	1.22-1.81	**<.001**
Tumor diameter, cm	1.01	0.95-1.08	.76	1.03	0.96-1.1	.36
Preoperative KPS						
100 - 80	Ref	Ref	Ref	Ref	Ref	Ref
≤70	0.97	0.74-1.28	.85	1.44	1.1-1.9	**.01**
Extent of resection						
GTR	0.6	0.5-0.73	**<.001**	0.7	0.57-0.85	**<.001**
STR	Ref	Ref	Ref	Ref	Ref	Ref
MGMT methylation	0.6	0.49-0.73	**<.001**	0.65	0.53-0.79	**<.001**
Chemoradiation	0.28	0.16-0.48	**<.001**	0.2	0.2-0.34	**<S.001**
Ischemic changes						
None or rim restriction	Ref	Ref	Ref	Ref	Ref	Ref
Small (<5 cm^3^)	0.94	0.68-1.31	.74	0.8	0.59-1.2	.26
Larger, confluent (≥ 5 cm³)	1.4	1.01-1.94	**.047**	1.7	1.2-2.4	**.001**
New or worsened deficits at discharge	1.38	1.1-1.8	**.01**	1.32	1.02-1.7	**.03**

Abbreviations: IDH, isocitrate dehydrogenase; KPS, Karnofsky Performance Scale; MGMT, O6-Methylguanine-DNA Methyltransferase.Bold text indicates statistical significance.

### Impact of Postoperative Ischemia on PFS and OS in Patients Undergoing GTR

Lastly, a separate Kaplan–Meier analysis was performed to assess and visualize the impact of postoperative ischemia on survival outcomes in patients achieving GTR.

**Figure 3.**
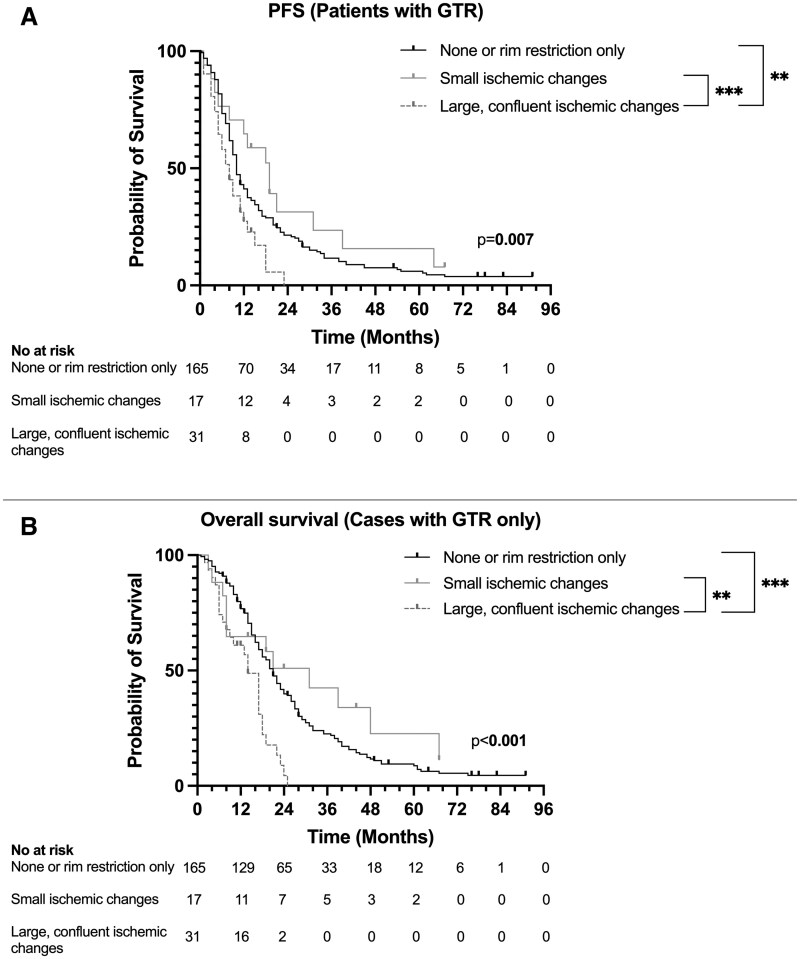
(A) Progression-free survival (PFS) in patients with GTR, stratified by the severity of ischemic changes. Large, confluent ischemic changes were associated with significantly shorter PFS compared to small ischemic changes (*P* = .02) and none/rim restriction only (*P* = .008). (B) Overall survival (OS) in patients with GTR, stratified by the severity of ischemic changes. Large, confluent ischemic changes were associated with significantly shorter OS compared to small ischemic changes (*P* = .009) and none/rim restriction only (*P* = .001).

The median PFS was 10 months [8.8-11.2] in patients with GTR. Patients with large ischemic changes had a significantly shorter PFS (8 months [5-12]) compared to those with small ischemic changes (19 months [12—not calculable], *P* = .008) and no ischemia or rim restriction only (10 months [9-12], *P* = .002), while no difference was observed between the small ischemic and no-ischemia groups (Figure 3A).

The median OS was 20 months [17.7-22.4] in patients with GTR. Similar to PFS, patients with large ischemic changes had the shortest OS (14 months [9-18]) compared to those with no ischemia or rim restriction only (21 months [18-24], *P* = .001) and those with small ischemic changes (31 months [8—not calculable], *P* = .009) (Figure 3B).

## Discussion

This study corroborates and extends prior research demonstrating a progressive decline in survival with increasing infarct volume, and further reveals that this deleterious association is most pronounced in the presence of large, confluent infarcts.^4^^,^^11^

Postoperative ischemic changes are not an uncommon occurrence after glioma surgery, with reported rates ranging between 25% to 70%.^4^^,^^7^^,^^8^^,^^11^^,^^13^^,^^15^^,^^18^ They usually reflect compromise of small perforating or peritumoral vessels supplying the adjacent white matter or thermal injury from coagulation near the cavity margin.^7^^,^^8^ Even with careful subpial dissection, vessel preservation, and functional mapping, particularly near eloquent areas, they are not always completely avoidable.^7^^,^^8^ In our series, ischemia beyond a thin rim restriction was present in 20.8% of patients, and more often in tumors involving the insula, temporal lobe, or basal ganglia, and in larger tumors. In line with previous studies, we did not observe an association between ischemic changes and age, smoking history, or preoperative KPS, suggesting that these changes are primarily related to the surgical resection itself.^7^^,^^19^

The impact of ischemia on survival outcomes in patients with glioma remains unclear. Several studies have reported an association between ischemic changes and neurological deficits, which have been shown to portend poorer survival in patients with glioma.^7^^,^^11^^,^^19^^,^^20^ In this study, we observed a moderate positive correlation between infarct volume and new or worsened deficits (*r* = 0.29, *P* < .001), indicating that although these deficits might often be associated with ischemic lesions, not all could be attributed to ischemia, and ischemia did not invariably lead to new deficits. Given these findings, we further examined the impact of ischemic changes on survival in multivariable analyses. which showed that large, confluent ischemic changes (>5 cm^3^) were associated with poorer survival, independent of new or worsened deficits. Although we assessed new or worsened neurological deficits at discharge, this measure may underestimate the true extent of functional impairment, and the retrospective nature of our study limited the depth of postoperative evaluations. Nearly half (42.9%) of large confluent ischemic lesions in our series involved eloquent cortex, where the resulting deficits are likely to have greater clinical impact. Van der Boog et al^7^ similarly noted that most patients with persistent deficits (beyond 3 months) in their cohort had large confluent ischemia. The potential long-term impact of these postoperative changes on quality of life and higher cognitive function remains uncertain, and future studies should examine whether they affect broader functional domains not captured by KPS or standard neurological examination, as such effects could still have meaningful implications for patient outcomes over time.

Similar to this study, Bette et al. reported a significant and independent association between infarct volume and reduced survival in GBM, attributing this effect to an increased incidence of multifocal and distant tumor recurrences.^11^^,^^16^ Thiepold et al^17^ also noted a higher rate of diffuse or distant recurrences in patients with perioperative ischemia compared with the control group; however, this was not associated with a difference in survival. Authors emphasized the critical role of hypoxia in GBM pathogenesis, particularly in driving invasion, therapeutic resistance, and metabolic reprogramming—processes primarily orchestrated by hypoxia-inducible factors (HIF-1α and HIF-2α).^21–24^ Authors concluded that postoperative infarct volume might initiate hypoxia-mediated aggressive tumor growth resulting in multifocal and diffuse recurrence patterns and impaired survival.^11^^,^^16^^,^^17^ This study suggests a similar difference in recurrence patterns (ischemic changes vs no ischemia; multifocal: 37.2% vs 31.9%; distant: 42.6% vs 37.8%), although the observed difference did not reach statistical significance. In contrast, van der Boog et al. found no correlation between infarct volume and survival in a mixed cohort of high- and low-grade gliomas, whereas Aaronson et al. reported that postoperative ischemic changes were significantly linked to reduced survival, specifically in high-grade glioma patients.^4^^,^^7^ While variability in study designs may account for these conflicting findings, differences in patient populations—particularly molecular profile—may also have played a role. A recent study demonstrated that IDH mutation status modulates glioma cell responses to hypoxia, with IDH–wildtype tumors exhibiting a shift toward a mesenchymal-like state associated with increased invasiveness, whereas IDH–mutant gliomas preferentially adopt an astrocyte-like phenotype, a transition that may contribute to a more favorable prognosis.^25^ These findings suggest that the impact of ischemia on survival may be shaped by the molecular profile of the tumor, though further investigation is warranted.

In our cohort, patients who underwent GTR but developed large postoperative ischemic changes had significantly shorter PFS and OS compared to those with no ischemia or with small ischemic changes. Notably, there was no significant difference in PFS and OS between patients with small ischemic changes and those without ischemia, suggesting that infarct burden may play a role in survival outcomes. In the era of emerging supramaximal resection strategies, it will be important to determine whether the observed benefit is partly related to the resection of ischemic tissue, and to clarify the role of this approach in patients who develop large ischemic changes. Overall, these findings underscore the importance of meticulous tumor resection, maximizing EOR while preserving functional outcomes and quality of life, so as not to attenuate or negate the intended therapeutic gain.

### Limitations

The findings of this study are subject to the inherent limitations of a retrospective, single-center design. Although adjustments were made for key molecular and clinical variables, the potential influence of unmeasured confounders and institutional variations in treatment protocols cannot be entirely excluded. Given the inclusion of cases diagnosed before the 2021 WHO classification update, some variability in IDH assessment may exist, although only tumors classified as IDH-wildtype based on available data were included. Nonetheless, the strength and consistency of the observed associations underscore the validity of the findings and their relevance to current clinical practice. Future prospective, multicenter studies will be essential to further validate these results and enhance their generalizability across diverse patient populations.

In conclusion, in this study, an increased volume of ischemic changes was found to be associated with reduced survival, independent of neurological function. These findings suggest that hypoxia may be an important mediator of disease progression in GBM. Understanding the cellular manifestations of hypoxia could serve as a basis for new therapeutic avenues and for refining surgical approaches to improve patient outcomes.

## Data Availability

The data supporting this study are confidential patient records and are not publicly available. They may be provided upon reasonable request, subject to institutional review board approval and data protection regulations.
